# Supplementary material for: Alterations in the Metabolic and Lipid Profiles Associated with Vitamin D Deficiency in Early Pregnancy
Source: Nutrients. 2025 Sep 29;17(19):3096. doi: 10.3390/nu17193096 (PMC12526048; doi:10.3390/nu17193096)
Supplement: Supplementary file 1 [file nutrients-17-03096-s001.zip › nutrients-3889173-supplementary.pdf]

Supplementary Table S1. Detailed information of VDD-associated metabolites.

FeatureID	Log_2FC	VIP	*P*	MS2.name	Super.Class	Class	Sub.Class
Chem_997	0.630	2.155	0.020	Bis(2,4,6-trimethylphenyl)phosphine	Benzenoids	Benzene and substituted derivatives	Benzene and substituted derivatives
Chem_975	0.494	2.787	0.002	N-Cyclohexylformamide	Benzenoids	Benzene and substituted derivatives	Benzene and substituted derivatives
Chem_694	0.450	1.900	0.041	5-Nitro-2-toluidine	Benzenoids	Benzene and substituted derivatives	Nitrobenzenes
Chem_236	0.242	1.967	0.034	2,5-Di-tert-butylhydroquinone	Benzenoids	Benzene and substituted derivatives	Phenylpropanes
Chem_584	-0.233	1.953	0.036	Butylated hydroxytoluene	Benzenoids	Benzene and substituted derivatives	Phenylpropanes
Chem_1145	-0.248	2.065	0.026	3-(3,4-Dimethoxyphenyl)-2-thioxo-1,3-thiazolidin-4-one	Benzenoids	Benzene and substituted derivatives	Methoxybenzenes
Chem_910	0.506	2.592	0.005	Methyl 1-hydroxy-2-naphthoate	Benzenoids	Naphthalenes	Naphthalenecarboxylic acids and derivatives
Chem_504	0.321	2.018	0.030	.alpha.-Hydroxymetoprolol	Benzenoids	Phenol ethers	Phenol ethers
Chem_926	-0.240	2.002	0.031	2-[(4-Amino-6-anilino-1,3,5-triazin-2-yl)methoxy]benzaldehyde	Benzenoids	Phenol ethers	Phenol ethers
Chem_210	0.400	2.142	0.021	Hexylresorcinol	Benzenoids	Phenols	Benzenediols
Chem_590	0.346	2.131	0.021	Nadolol	Benzenoids	Tetralins	Tetralins
Chem_719	0.337	1.845	0.048	Octenoyl-carnitine	Lipids and lipid-like molecules	Fatty Acyls	Fatty acid esters
Chem_766	0.333	2.727	0.003	Hexenoylcarnitine (Car(6:1))	Lipids and lipid-like molecules	Fatty Acyls	Fatty acid esters
Chem_788	-0.313	2.117	0.022	9Z,11E,13E-Octadecatrienoic acid	Lipids and lipid-like molecules	Fatty Acyls	Lineolic acids and derivatives
Chem_1109	-0.388	2.084	0.025	10E,12Z-octadecadienoic acid	Lipids and lipid-like molecules	Fatty Acyls	Lineolic acids and derivatives
Chem_1022	-0.411	1.942	0.038	DG(18:2(9Z,12Z)/15:0/0:0)	Lipids and lipid-like molecules	Fatty Acyls	Lineolic acids and derivatives
Chem_1197	-0.554	2.229	0.016	12,13-Dihydroxy-9Z-octadecenoic acid	Lipids and lipid-like molecules	Fatty Acyls	Fatty acids and conjugates
Chem_1200	0.439	2.089	0.025	PC(20:4(8Z,11Z,14Z,17Z)/15:0)	Lipids and lipid-like molecules	Glycerophospholipids	Glycerophosphocholines

Chem_785	-0.244	1.986	0.032	1,2-Di-(9Z,12Z,15Z-octadecatrienoyl)-sn-glycero-3-phosphocholine	Lipids and lipid-like molecules	Glycerophospholipids	Glycerophosphocholines
Chem_432	-0.275	2.023	0.030	PC(P-18:0/18:1(9Z))	Lipids and lipid-like molecules	Glycerophospholipids	Glycerophosphocholines
Chem_739	-0.276	2.396	0.009	PC(32:2)	Lipids and lipid-like molecules	Glycerophospholipids	Glycerophosphocholines
Chem_129	-0.278	2.234	0.016	Glycerophosphoethanolamine	Lipids and lipid-like molecules	Glycerophospholipids	Glycerophosphoethanolamines
Chem_1165	-0.283	2.466	0.007	PC(20:3(5Z,8Z,11Z)/14:1(9Z))	Lipids and lipid-like molecules	Glycerophospholipids	Glycerophosphocholines
Chem_256	-0.307	2.352	0.011	1-Myristoyl-sn-glycero-3-phosphocholine (LPC(14:0/0:0))	Lipids and lipid-like molecules	Glycerophospholipids	Glycerophosphocholines
Chem_1158	-0.309	1.925	0.039	PC(16:0/16:1(9Z))	Lipids and lipid-like molecules	Glycerophospholipids	Glycerophosphocholines
Chem_392	-0.342	1.865	0.045	PC(14:0/16:0)	Lipids and lipid-like molecules	Glycerophospholipids	Glycerophosphocholines
Chem_594	0.497	2.151	0.021	Perillic_acid	Lipids and lipid-like molecules	Prenol lipids	Monoterpenoids
Chem_831	-0.385	2.017	0.031	(5E,9E)-Farnesylacetone	Lipids and lipid-like molecules	Prenol lipids	Diterpenoids
Chem_1199	-0.402	2.366	0.011	Lepidiumterpenoid	Lipids and lipid-like molecules	Prenol lipids	Diterpenoids
Chem_983	0.337	2.056	0.027	Androstan-4,6-diene-17.beta.-ol-3-one	Lipids and lipid-like molecules	Steroids and steroid derivatives	Androstane steroids
Chem_1251	-0.446	2.276	0.014	Ergostane-3,6-dione	Lipids and lipid-like molecules	Steroids and steroid derivatives	Ergostane steroids
Chem_233	-0.879	2.382	0.011	Lithocholic acid 3-sulfate	Lipids and lipid-like molecules	Steroids and steroid derivatives	Bile acids, alcohols and derivatives
Chem_1146	0.527	1.857	0.047	3-[5-(2-Methylpropyl)-3,6-dioxopiperazin-2-yl]propanoic acid	Organic acids and derivatives	Carboximidic acids and derivatives	Carboximidic acids
Chem_1167	0.452	2.708	0.003	Ser-Glu	Organic acids and derivatives	Carboxylic acids and derivatives	Amino acids, peptides, and analogues
Chem_467	0.363	2.777	0.003	Dimethylmalonic acid	Organic acids and derivatives	Carboxylic acids and derivatives	Dicarboxylic acids and derivatives
Chem_874	0.361	2.492	0.007	L-Arginine, methyl ester	Organic acids and derivatives	Carboxylic acids and derivatives	Amino acids, peptides, and analogues
Chem_716	0.288	1.840	0.048	Creatine, ethyl ester	Organic acids and derivatives	Carboxylic acids and derivatives	Amino acids, peptides, and analogues

Chem_157	0.869	2.353	0.012	3-Hydroxybutyric acid	Organic acids and derivatives	Hydroxy acids and derivatives	Beta hydroxy acids and derivatives
Chem_1156	-2.271	2.233	0.018	Mevinic acid	Organic acids and derivatives	Hydroxy acids and derivatives	Medium-chain hydroxy acids and derivatives
Chem_1196	0.635	2.896	0.002	3-Oxocyclobutanecarboxylic acid	Organic oxygen compounds	Organooxygen compounds	Carbonyl compounds
Chem_929	0.502	3.642	0.000	1-Deoxy-D-glucitol	Organic oxygen compounds	Organooxygen compounds	Carbohydrates and carbohydrate conjugates
Chem_9	0.453	3.174	0.000	Pantothenic acid	Organic oxygen compounds	Organooxygen compounds	Alcohols and polyols
Chem_1414	0.226	2.084	0.025	ACARBOSE	Organic oxygen compounds	Organooxygen compounds	Carbohydrates and carbohydrate conjugates
Chem_32	-0.363	1.967	0.035	Tartaric acid	Organic oxygen compounds	Organooxygen compounds	Carbohydrates and carbohydrate conjugates
Chem_1428	-0.440	2.220	0.017	Thiothixene	Organoheterocyclic compounds	Benzothiopyrans	1-benzothiopyrans
Chem_1306	0.302	1.888	0.043	Ethyl 6-methyl-4-(4-methylphenyl)-2-oxo-1,2,3,4-tetrahydro-5-pyrimidinecarboxylate	Organoheterocyclic compounds	Diazines	Pyrimidines and pyrimidine derivatives
Chem_629	0.186	1.879	0.043	4-Cyanotetrahydropyran-4-carboxylic acid	Organoheterocyclic compounds	Oxanes	Oxanes
Chem_29	0.463	2.713	0.003	N1-Methyl-2-pyridone-5-carboxamide	Organoheterocyclic compounds	Pyridines and derivatives	Pyridinecarboxylic acids and derivatives
Chem_300	0.463	2.713	0.003	N1-Methyl-4-pyridone-3-carboxamide	Organoheterocyclic compounds	Pyridines and derivatives	Pyridinecarboxylic acids and derivatives
Chem_675	0.432	2.036	0.028	Ethosuximide	Organoheterocyclic compounds	Pyrrolidines	Pyrrolidones
Chem_112	1.117	1.989	0.035	NA	NA	NA	NA
Chem_418	1.117	1.989	0.035	NA	NA	NA	NA
Chem_434	1.117	1.989	0.035	NA	NA	NA	NA
Chem_731	0.732	2.680	0.004	Octenedioylcarnitine (Car(8:1-O2))	NA	NA	NA
Chem_271	0.265	1.893	0.042	NA	NA	NA	NA
Chem_420	0.265	1.893	0.042	NA	NA	NA	NA
Chem_606	-0.702	1.899	0.041	NA	NA	NA	NA
Chem_20	-0.763	2.282	0.014	NA	NA	NA	NA

Chem_1113	-0.807	2.155	0.022	NA	NA	NA	NA
Chem_107	-0.872	1.858	0.047	NA	NA	NA	NA

FC, fold change; VIP, variable importance in projection.
